# Comparative Analysis of Physiological Parameters, Antioxidant Defense, Ion Regulation, and Gene Expression in Two Distinct Maize Hybrids Under Salt Stress at Seedling Stage

**DOI:** 10.3390/life15040591

**Published:** 2025-04-03

**Authors:** Medhat Rehan, Mohamed M. Kamara, Hassan Barakat

**Affiliations:** 1 Department of Plant Production, College of Agriculture and Food, Qassim University, Buraydah 51452, Saudi Arabia; 2 Department of Agronomy, Faculty of Agriculture, Kafrelsheikh University, Kafr El-Sheikh 33516, Egypt; mohamed.kamara@agr.kfs.edu.eg; 3 Department of Food Science and Human Nutrition, College of Agriculture and Food, Qassim University, Buraydah 51452, Saudi Arabia

**Keywords:** salt tolerance, antioxidant, ion regulation, proline accumulation, Na^+^ to K^+^ ratio, gene expression, osmotic adjustment, stress resilience, sustainability

## Abstract

Salinity significantly impacts maize production globally, requiring a deeper understanding of maize response mechanisms to salt stress. This study assessed the response of two Egyptian maize hybrids, SC-10 and TWC-321, under salt stress (200 mM NaCl) and non-stressed conditions to identify traits and mechanisms linked to enhanced salinity tolerance. Both hybrids accumulated similar Na^+^ levels in leaves, but TWC-321 exhibited better ion regulation, with lower Na^+^ concentrations and Na^+^ to K^+^ ratio in roots. While SC-10 showed a reduction in leaf K^+^ levels, TWC-321 maintained stable K^+^ levels, highlighting its superior salinity tolerance. TWC-321 also demonstrated better oxidative stress management, as evidenced by lower malondialdehyde levels and significantly higher total chlorophyll content, relative water content, and stomatal conductance. Proline accumulation was more pronounced in TWC-321, and it showed higher antioxidant enzyme activities (SOD, CAT, and POD) compared to SC-10, which exhibited lower SOD and POD activities. Gene expression analysis demonstrated distinct responses to salt stress between the hybrids. Although *zmHKT1;5* was similarly induced in both hybrids, TWC-321 exhibited higher expression levels of *zmHKT2* (1.96-fold compared to 1.42-fold in SC-10) and upregulated *zmNHX1* (1.92-fold), whereas *zmNHX1* expression was slightly reduced in SC-10 (0.8-fold). Additionally, TWC-321 achieved a greater total dry weight than SC-10 under salinity stress, highlighting its superior performance and resilience. These findings indicate that enhanced Na^+^ exclusion and sequestration mechanisms mediate the salinity tolerance of TWC-321. Correlation analysis under salinity stress identified key indicators of salinity tolerance, including increased activity of CAT and SOD, elevated proline accumulation, and higher K^+^ content. Consequently, the salinity tolerance of TWC-321 can be attributed to its effective ion regulation, stable photosynthetic pigment levels, improved osmotic adjustment, enhanced water retention, and potent antioxidant defense system. These insights are highly valuable for breeding programs focused on developing salt-tolerant maize hybrids.

## 1. Introduction

Salinity is a widespread abiotic stress that severely affects plant development, growth, and survival, causing significant yield reductions [1,2]. Over 800 million hectares are adversely impacted by salinization, posing an essential global concern for arable land [3]. The expansion of salinized farmland is driven by factors such as climate changes, terrain, inefficient irrigation, and specific fertilization practices [4,5]. Salinity stress imposes both ionic and osmotic stresses [6,7], leading to various adverse effects such as membrane disruption, metabolic imbalance, and oxidative damage [8,9]. High soil salt concentrations cause osmotic stress, which impairs plant capacity to absorb water, causing physiological issues like decreased cell expansion and stomatal conductance [10,11]. This stress also negatively impacts photosynthesis, biomass accumulation, and yield, while impairing the uptake of essential nutrients [12,13]. Ionic toxicity occurs when sodium ion (Na^+^) concentrations rise in plant cells, particularly in the leaves, disrupting Na^+^ to K^+^ homeostasis and hindering growth and development [14]. Excess sodium disrupts cellular metabolism and causes ionic imbalance [15,16]. Additionally, it stimulates the generation of ROS, which cause oxidative damage to nucleic acids, lipids, and proteins, ultimately leading to programmed cell death [17,18].

Maize (*Zea mays*) is one of the most widely cultivated and economically significant cereal crops globally. It serves as a staple food source, livestock feed, and industrial raw material in many regions [19]. Maize is crucial to food security, particularly in developing countries, where it is a primary source of calories and essential nutrients [20]. However, maize cultivation is increasingly threatened by abiotic stresses, particularly salinity, which is a major environmental challenge in irrigated and rainfed areas [21]. Salinity affects maize at various growth stages, especially during the early seedling phase, leading to reduced germination, impaired root development, and decreased photosynthesis, ultimately resulting in yield losses [12]. Despite its economic importance, maize is highly sensitive to salinity, making it an important subject for research aimed at identifying and developing more tolerant varieties [22].

Under salt stress, plants adopt various strategies that involve hormone regulation, ion homeostasis, ROS detoxification, and osmotic balance [23,24]. To cope with osmotic stress, the plants minimize water loss and enhance the Na^+^ to K^+^ ratio [25]. Improving proline, soluble carbohydrates, and potassium (K^+^) promotes ion compartmentalization and inhibits the uptake of Na [26]. K^+^ is the principal component for regulating osmotic pressure and other cellular functions, making balance between K^+^ and Na^+^ critical for plant growth under salinity stress [27]. To provide a steady cytosolic Na^+^ to K^+^ ratio and reduce Na^+^ toxicity, plants either sequester or exclude Na^+^ from their leaves in response to ionic stress [28,29]. This mechanism is regulated by tonoplast Na^+^/H^+^ antiporters [30]. Furthermore, ROS are detoxified by antioxidant enzymes such as peroxidase (POD), superoxide dismutase (SOD), and catalase (CAT) [31]. SOD converts superoxide anions to hydrogen peroxide (H_2_O_2_), which is then detoxified by CAT, POD, and other enzymes to produce water (H_2_O) and oxygen (O_2_) [18]. Enhancing antioxidant activity mitigates ROS-induced damage and supports ion transport systems, which are crucial for maintaining the Na^+^ to K^+^ ratio [32,33,34].

Many plant species possess genes that contribute to improved salt tolerance. High-affinity potassium transporter (HKT) genes, which belong to the Trk/Ktr/HKT transporter family, play a crucial role in regulating the movement of K^+^ and Na^+^ in higher plants [35]. These transporters help remove Na^+^ from the xylem and transfer it into parenchyma cells, facilitating the movement of Na^+^ from roots to shoots [14]. *zmHKT1;5* encodes a high-affinity potassium transporter in maize that prevents Na^+^ from entering the xylem, reducing ionic toxicity and preventing Na^+^ from reaching the plant’s aerial parts [17]. Additionally, numerous alleles of HKT genes have been identified that give high degrees of salt stress tolerance in maize, and these alleles could be exploited in breeding programs to develop salt-tolerant hybrids [36]. Vacuolar membrane-localized Na^+^/H^+^ exchangers (NHXs) and HKT transporters are essential for salt tolerance. A balanced cytosolic Na^+^ to K^+^ ratio and optimal cellular activities under salt stress depend on vacuolar Na^+^ sequestration, a crucial function of *zmNHX1* [37]. By compartmentalizing excess Na^+^ into vacuoles, *zmNHX1* mitigates ionic toxicity and supports metabolic processes essential for plant growth and productivity. Understanding the expression of these genes is critical for clarifying the mechanisms of salinity tolerance and creating genetically improved genotypes that can grow in saline environments.

The integration of physiological, biochemical, and molecular responses is crucial for the identification of salt-sensitive and salt-tolerant genotypes [38]. Laboratory-based tests offer precise control over salt concentrations, enabling accurate data collection and making them particularly valuable for assessing salt tolerance during the early seedling stage [39]. Understanding how maize responds to salinity stress is key to developing resilient hybrids [40]. In Egypt, the SC-10 and TWC-321 maize hybrids are widely cultivated due to their stability across diverse environmental conditions and resistance to major diseases [41]. However, the response of these hybrids to salinity remains unexplored, especially in the early development stage. Hence, the objectives of this study were to compare the physiological responses of two distinct maize hybrids under salt stress, focusing on ion regulation (Na^+^ and K^+^ concentrations and Na^+^ to K^+^ ratio), chlorophyll content, photosynthetic efficiency, water retention, proline accumulation, oxidative stress markers (MDA), and membrane stability. Additionally, this study aimed to evaluate antioxidant enzyme activity (SOD, POD, and CAT), gene expression of HKT transporters and NHX antiporters, and growth parameters (root and shoot length, leaf area, and dry weight). This study also uses principal component analysis and heatmap clustering to identify key traits linked to salt tolerance and explores the correlations between physiological and biochemical responses under salinity stress.

## 2. Materials and Methods

### 2.1. Plant Materials and Growth Conditions

The Field Crops Research Institute in Egypt provided seeds of two Egyptian maize hybrids, SC-10 and TWC-321. These hybrids were chosen due to their widespread cultivation across Egypt, where they are recognized for being high-yielding, adaptable to various environmental conditions, and resistant to major diseases. The seeds of both hybrids were surface sterilized for 30 min in a sodium hypochlorite solution (5.0%) before being rinsed with distilled water. Subsequently, the seeds were planted in plastic pots (30 cm in depth and 20 cm in diameter) filled with previously cleaned sand, with five seeds per pot. Then, the pots were thinned, and only one seedling per pot was retained. Each pot was irrigated every two days using a half-strength Hoagland solution [42]. The pots were maintained in a growth chamber at 24 ± 2 °C with a 16 h photoperiod, a photosynthetic photon flux density of 180 µmol m^−2^ s^−1^, and 70% relative humidity. Two primary sets of uniformly sized two-week-old seedlings were created. The first group was consistently irrigated with a half-strength Hoagland solution with 0 mM NaCl (non-stress control). The other group was given a half-strength Hoagland solution supplemented with 200 mM NaCl to induce salinity stress. The salt treatment was given gradually at an interval of 2 days to avoid membrane damage from osmotic shock, and the start of the salt (NaCl) treatment was considered when the desired concentration of 200 mM NaCl was added to the salt-treated plants. Four replicates of a Completely Randomized Design (CRD) were applied in this experiment. The plants were left to develop for ten days, and then the seedlings were harvested for molecular, biochemical, mineral, and growth analysis.

### 2.2. Na^+^ and K^+^ Concentration Measurements

The roots and leaves were finely ground after being oven-dried. A flame photometer (ELEX 6361, Hamburg, Germany) was used to quantify the amounts of Na^+^ and K^+^ in the extracts following the digestion of the resultant powder with a mixture of HNO_3_ and HClO_4_ (5:1, *v*/*v*) as illustrated by Wolf [43]. The concentrations of Na^+^ and K^+^ are expressed in milligrams per gram of dry weight (mg/g DW). The Na^+^ to K^+^ ratio was calculated by dividing the Na^+^ concentration by the K^+^ concentration.

### 2.3. Chlorophyll and Photosynthetic Parameter Measurements

Leaf samples (0.5 g) were centrifuged at 10,000× *g* for 10 min after being homogenized in cold 85% acetone at 4 °C in a dark environment. At 663 and 646 nm, the absorbances of the supernatants were measured with a UV–visible spectrophotometer (Spectra-Max-Plus, San Jose, CA, USA). The equations of Lichtenthaler and Buschmann [44] were used to determine the levels of chlorophyll *a*, chlorophyll *b*, and total chlorophyll. The results are expressed in milligrams per gram of fresh weight (mg/g FW). An LI-6400-portable-photosynthetic system (Li-COR, Lincoln, NE, USA) was placed on the second completely grown leaf from the top to assess the transpiration rate and stomatal conductance. The transpiration rate is expressed in micromoles of water vapor per square meter per second (µmol/m^2^/s), and stomatal conductance is expressed in millimoles of CO_2_ per square meter per second (mmol/m^2^/s).

### 2.4. Relative Water Content (RWC) Measurement

The approach outlined by Barrs and Weatherley [45] was utilized to determine RWC. First, the leaf samples’ fresh weight (FW) was determined by direct weighing. To obtain complete turgidity, the samples were immersed in fresh deionized water for a day in a light environment. Following soaking, the samples were weighed to ascertain their turgid weight (TW) and blotted with tissue paper to remove any remaining surface water. The samples were then oven-dried for three days at 70 °C, and their dry weight (DW) was determined. The RWC (%) was calculated by following the formula (FW − DW)/(TW − DW) × 100.

### 2.5. Proline Measurement

Using 0.5 g of leaf tissue, the proline concentration was recorded following the procedure of Bates et al. [46]. After homogenizing the leaf samples in sulfosalicylic acid, two milliliters of the extract were put in a glass tube with two milliliters of acid-ninhydrin. The mixture was incubated at 100 °C in a water bath for one hour. Following incubation, the samples were allowed to cool before being submerged in ice water, and then 4 milliliters of toluene was added. To ensure proper blending, the concoction was shaken vigorously. The absorbance at 560 nm of the top layer containing the chromophore was then measured. Proline content was determined using a standard curve of L-proline concentrations and is expressed in micromoles of proline per gram of fresh weight (µmol/g FW).

### 2.6. Membranes Stability Index (MSI) and Malondialdehyde (MDA)

Leaf samples were soaked in deionized water on a shaker for 12 h to determine MSI. A portable electrical conductivity meter (CM-21P; DKK-TOA-Corporation, Tokyo, Japan) was used to test initial electrical conductivity (EC_1_). To determine final electrical conductivity (EC_2_), the samples were autoclaved for 20 min at 120 °C. The following formula was used to determine MSI: MSI = (1 − (EC1/EC2)) × 100. MSI is expressed as a percentage (%) and reflects the integrity of the plant cell membranes. The method outlined by Du and Bramlage [47] was used to measure the MDA content. After 500 mg of fresh tissue was homogenized in 20% thiobarbituric acid with 0.01% butylated hydroxytoluene, it was incubated at 95 °C for 10 min before being centrifuged at 10,000× *g*. Absorbance at 532 nm and 600 nm was measured to determine MDA levels. MDA content was expressed in micromoles per gram of fresh weight (nmol/g FW).

### 2.7. Antioxidant Enzyme Identification (CAT, SOD, and POD)

Leaf tissue (0.5 g) was ground and mixed with 4 mL of sodium phosphate buffer (pH 7.0), which contained 0.1 mM EDTA and 1% (*w*/*v*) polyvinylpyrrolidone (PVP). The enzyme extract was obtained by centrifuging the homogenate at 10,000 rpm for 15 min at 4 °C. Enzyme activities were measured in the resulting supernatant [48]. Catalase (CAT) activity was determined following the method of Aebi [49] by measuring the decrease in absorbance at 240 nm due to the breakdown of H_2_O_2_. The reaction mixture contained 50 mM sodium phosphate buffer (pH 7.0), 10 mM H_2_O_2_, and 0.1 mL of enzyme extract. The activity is expressed as units per milligram of protein (U/mg protein). Peroxidase (POD) activity was measured using the method of Hammerschmidt et al. [50], where the reaction mixture consisted of 50 mM sodium phosphate buffer (pH 7.0), 50 μM guaiacol, and 10 mM H_2_O_2_. The change in absorbance at 470 nm was recorded, and results are expressed as units per milligram of protein (U/mg protein), where one unit corresponds to a change in absorbance of 0.01 per minute. Superoxide dismutase (SOD) activity was determined using the method of Giannopolitis and Ries [51], where the reaction mixture contained 50 mM sodium carbonate buffer (pH 10.2), 0.1 mM EDTA, 12.5 μM of nitroblue tetrazolium (NBT), and 50 μM of riboflavin. The activity was measured by the decrease in absorbance at 560 nm, and results are expressed as units per milligram of protein (U/mg protein), where one unit corresponds to the amount of enzyme that inhibits the reduction in NBT by 50%.

### 2.8. Analysis of Gene Expression

Total RNA was extracted from leaves of both stressed and non-stressed plants using TRIzol™ LS Reagent (Invitrogen, Carlsbad, CA, USA), following the manufacturer’s instructions. The extracted RNA (1 µg) was reverse-transcribed into complementary DNA (cDNA) using the SuperScript IV VILO Master Mix-Kit (Cat.No. 11766050, Invitrogen) after treatment with Dnase I (ezDNase, Invitrogen). Quantitative real-time PCR (qRT-PCR) was performed on the Applied Biosystems Real-Time PCR System (7500) using the QuantiTect SYBR Green PCR Kit (Cat. No. 204143, Qiagen, Hilden, Germany). The qPCR cycling conditions included initial denaturation at 95 °C for 15 min, followed by 40 cycles of denaturation at 94 °C for 15 s, annealing at 55 °C for 30 s, and extension at 72 °C for 30 s. The actin gene was used as the reference gene, and its stability under both stress and non-stress conditions was verified prior to use. Gene expression levels were calculated using the comparative 2^−ΔΔCT^ method as described by Livak and Schmittgen [52] from three biological replicates. Primer sequences used in the expression analysis are listed in Table 1.

### 2.9. Determination of Growth Parameters

At the end of treatments, the roots and shoots were separated, and a ruler was used to measure root length (RL) and shoot length (SL) in centimeters. Additionally, after the plant was oven-dried for 72 h at 70 °C, the total dry weight in grams of the entire plant (shoot and root) was measured. A LI-3000A Portable Leaf Area Meter (LI-COR Biosciences, Lincoln, NE, USA) was used to calculate the leaf area (LA).

### 2.10. Data Analysis

SPSS statistical software (version 22) was used to perform a one-way analysis of variance (ANOVA) on the collected data. The Tukey Honest Significant Difference (HSD) test was used to compare mean values at a significance level of *p* < 0.01. Principal component analysis (PCA) was carried out using the Facto-Extra package, and a heatmap was produced using the ComplexHeatmap package in R programming (version 4.0.3).

## 3. Results

### 3.1. Na^+^ and K^+^ Concentrations and Na^+^ to K^+^ Ratio

**Figure 1:**
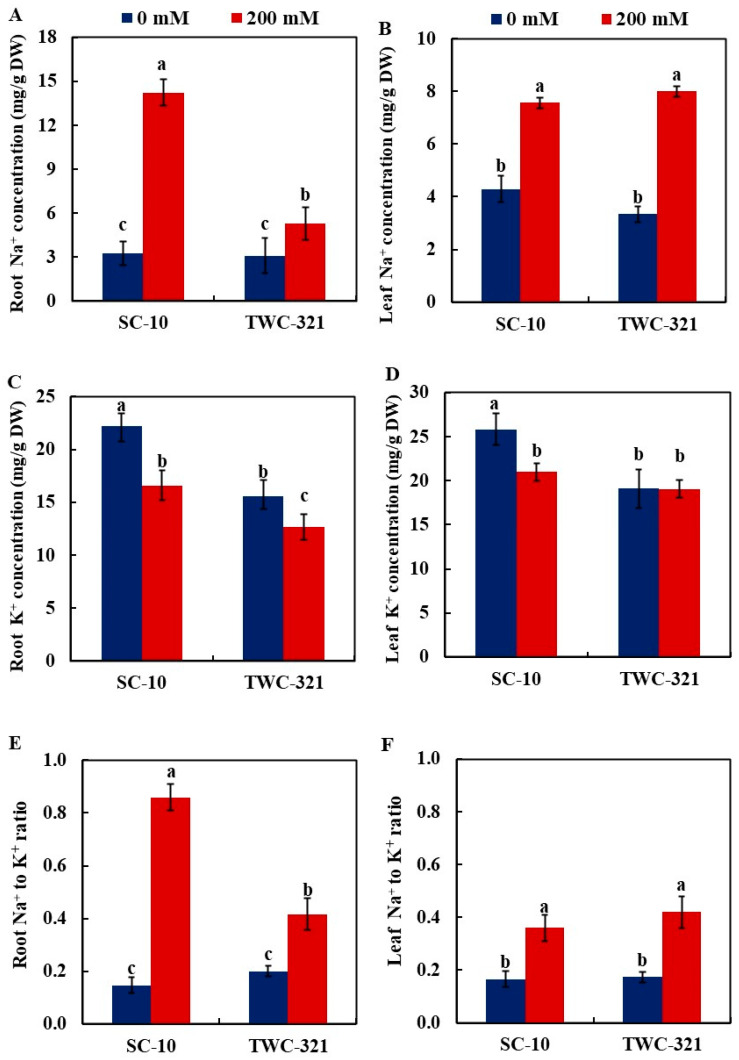
Effect of salt stress on root Na^+^ concentration (**A**), leaf Na + concentration (**B**), root K^+^ concentration (**C**), leaf K^+^ concentration (**D**), root Na^+^ to K^+^ (**E**), and leaf Na^+^ to K^+^ (**F**) of maize hybrids SC-10 and TWC-321. The data represent the average of 4 replicates ± SE. Significant differences are shown by the different letters on the columns (*p* < 0.01).

In comparison to the non-stressed control, salinity stress significantly increased Na^+^ concentration in the roots and leaves of both hybrids. The concentration of Na^+^ in the roots of SC-10 was 2.7-fold that of TWC-321 under salt stress (Figure 1A). Nonetheless, there was no significant difference in leaf Na^+^ contents of the two hybrids under salt stress (Figure 1B). Conversely, the salt treatment reduced K^+^ content in the roots of both hybrids, but no significant difference was observed in the leaves of TWC-321 between the control and stress treatments. Under salt stress, the K^+^ concentration in roots was reduced by 25.3% in SC-10 and 18.27% in TWC-321 compared to control conditions (Figure 1C). Similarly, K^+^ content in leaves decreased by 18.74% in SC-10 but remained unchanged in TWC-321 (Figure 1D). The root Na^+^ to K^+^ ratio increased two-fold in SC-10 compared to TWC-321 (Figure 1E). Under control conditions, there were no significant variations in the root Na^+^ to K^+^ ratio of both hybrids. The leaf Na^+^ to K^+^ ratio did not differ substantially between the two hybrids under salt stress (Figure 1F). The Na^+^ to K^+^ ratio of the two hybrids did not significantly change under control conditions.

### 3.2. Chlorophyll Content and Photosynthetic Efficiency

**Figure 2:**
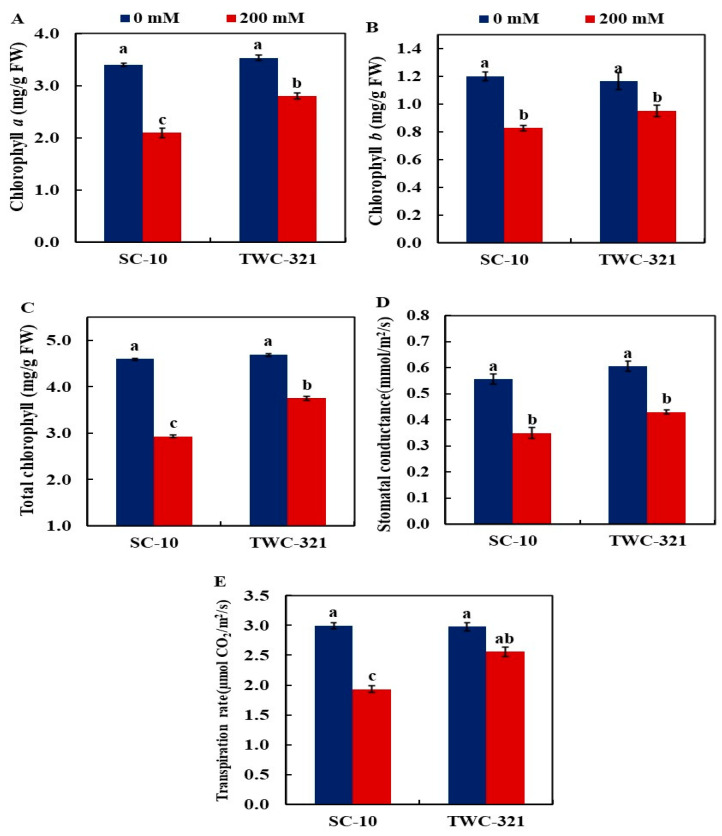
Impact of salt stress on chlorophyll a (**A**), chlorophyll b (**B**), total chlorophyll (**C**), stomatal conductance (**D**), and transpiration rate (**E**) of the maize hybrids SC-10 and TWC-321. The data represent the average of 4 replicates ± SE. Significant differences are shown by the different letters on the columns (*p* < 0.01).

Chlorophyll *a* and *b*, and total chlorophyll, exhibited significant reductions in SC-10, decreasing by 38.21%, 27.42%, and 36.34%, respectively (Figure 2A–C). In contrast, the reductions observed in TWC-321 were less pronounced, with decreases of 20.79%, 18.28%, and 20.17% in the same order, compared with the control treatment. The stomatal conductance under salt stress treatment decreased significantly in both hybrids, with SC-10 showing a more significant reduction (37.1%) than TWC-321 (24.1%) (Figure 2D). Similarly, the transpiration rate in SC-10 leaves decreased markedly, by 35.60%, under salt stress compared to the control, whereas in TWC-321, it showed only a slight reduction of 14.15% under the same conditions (Figure 2E).

### 3.3. Relative Water Content, Proline Accumulation, Malondialdehyde, and Membrane Stability Index

**Figure 3:**
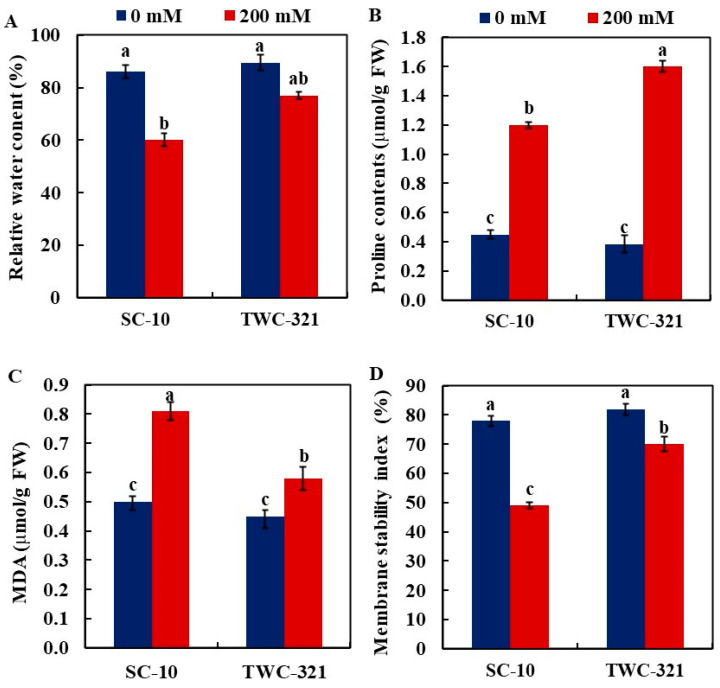
Influence of salt stress on relative water content (**A**), proline content (**B**), malondialdehyde (MDA) (**C**), and membrane stability index (**D**) of the maize hybrids SC-10 and TWC-321. The data represent the average of 4 replicates ± SE. Significant differences are shown by the distinct letters on the columns (*p* < 0.01).

Relative water content (RWC) was measured to assess water loss under salinity conditions. The RWC significantly decreased in SC-10 under salt stress (30.23%) compared to the control, whereas RWC did not differ substantially in TWC-321 under salt stress (Figure 3A). These results revealed that TWC-321 demonstrated a stronger ability to retain tissue water than SC-10. Proline accumulation in leaves of both hybrids was significantly increased by salinity stress; TWC-321 leaves increased by 4.2-fold, while SC-10 leaves increased by 2.7-fold (Figure 3B). The MDA concentration in leaves increased in both hybrids, but it was higher in SC-10 (62.0%) than in TWC-321 (28.89%) in comparison to the control (Figure 3C). Salt treatment significantly decreased the membrane stability index (MSI) in the leaves of both hybrids compared to the control treatment. However, the decrease was more marked in SC-10 (37.18%) than in TWC-321 (14.63%) (Figure 3D).

### 3.4. Activity of Antioxidant Enzymes

**Figure 4:**
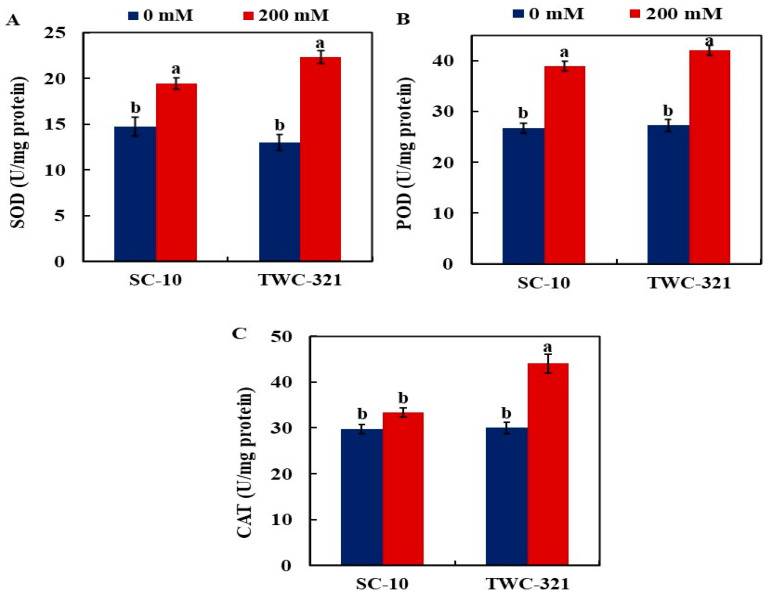
Effect of salt stress on the antioxidant enzymes superoxide dismutase (SOD) (**A**), peroxidase (POD) (**B**), and catalase (CAT) (**C**) of the maize hybrids SC-10 and TWC-321. The data represent the average of 4 replicates ± SE. Significant differences are shown by the distinct letters on the columns (*p* < 0.01).

The activity of the antioxidant enzymes SOD, POD, and CAT are presented in Figure 4A–C. Leaves of both hybrids showed a considerable increase in SOD activity under salinity treatment, with TWC-321 showing a comparatively larger response (61.33%) than SC-10 (28.2%) in comparison to controls (Figure 4A). POD activity was significantly elevated in both hybrids, while its activity was more significant in TWC-321 (54%) than in SC-10 (45% increase), compared with controls (Figure 4B). CAT activity remained statistically unaltered in SC-10, but the activity significantly increased in TWC-321 (46.33% increase) compared to the control (Figure 4C).

### 3.5. Gene Expression Analysis

**Figure 5:**
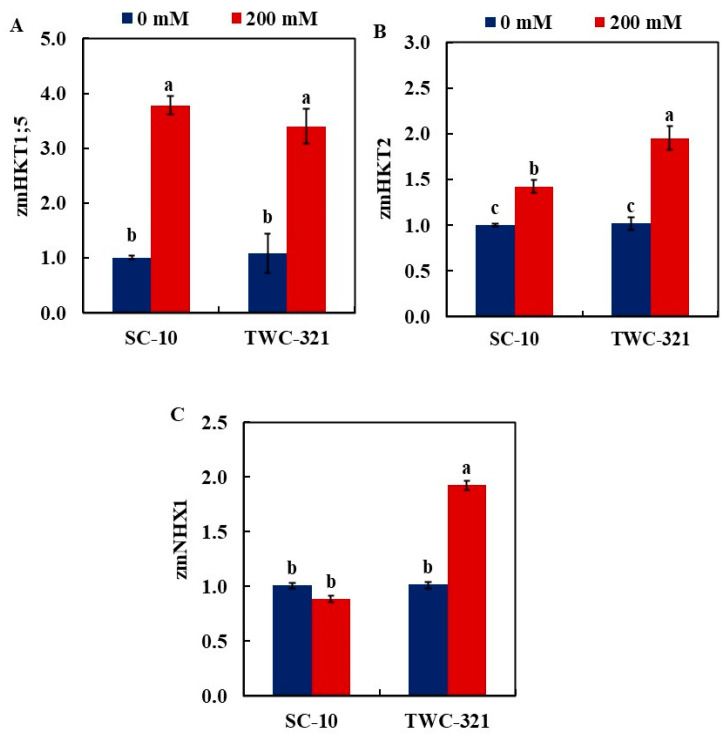
Impact of salt stress on gene expression in maize leaves: *zmHKT1;5* (**A**), *zmHKT2* (**B**), and *zmNHX1* (**C**). Data represent changes in expression levels under control and salinity conditions. Significant differences are shown by the distinct letters on the columns (*p* < 0.01).

Gene expression analysis of HKT transporters and NHX antiporters was conducted to investigate their contribution to salinity tolerance in maize. Quantitative RT-PCR analysis displayed that the salt stress treatment induces the expression of *zmHKT1;5* in the leaves of both SC-10 (3.78-fold) and TWC-321 (3.40-fold), with no significant differences between them (Figure 5A). The induction of *zmHKT2* in the leaves of TWC-321 (1.96-fold) was higher than that of SC-10 (1.42-fold) (Figure 5B). In TWC-321, salt stress increased *zmNHX1* gene expression by 1.92-fold, whereas in SC-10, it slightly decreased by 0.8-fold (Figure 5C).

### 3.6. Plant Growth

**Figure 6:**
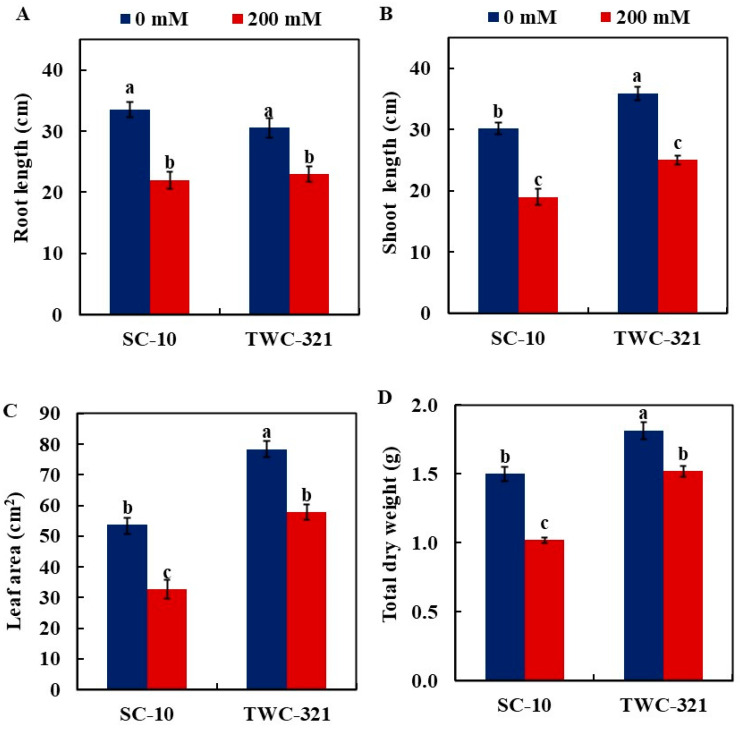
Response of root length (**A**), shoot length (**B**), leaf area (**C**), and dry weight (**D**) of maize hybrids SC-10 and TWC-321 to salt stress. The data represent the average of 4 replicates ± SE. Significant differences are shown by the distinct letters on the columns (*p* < 0.01).

Salinity stress decreased the growth of both hybrids; however, the decline was more remarkable in SC-10 than in TWC-321 (Figure 6). The reduction in root length was more pronounced in SC-10 (33.33%) compared to SC168 (24.74%). A similar pattern was observed for shoot length, with a 37.0% decrease in SC-10 and 29.8% in TWC-321 relative to the control plants (Figure 6A,B). Also, under salt stress, the leaf area of both hybrids decreased compared to controls (Figure 6C); however, the decline of TWC-321 was lower (26.3%) than that of SC-10 (38.94%). Moreover, the salt treatment decreased the total dry weights of SC-10 by 32.0% compared to 16.25% in TWC-321, compared to the controls (Figure 6D).

### 3.7. Principal Component and Heat Map Analyses

**Figure 7:**
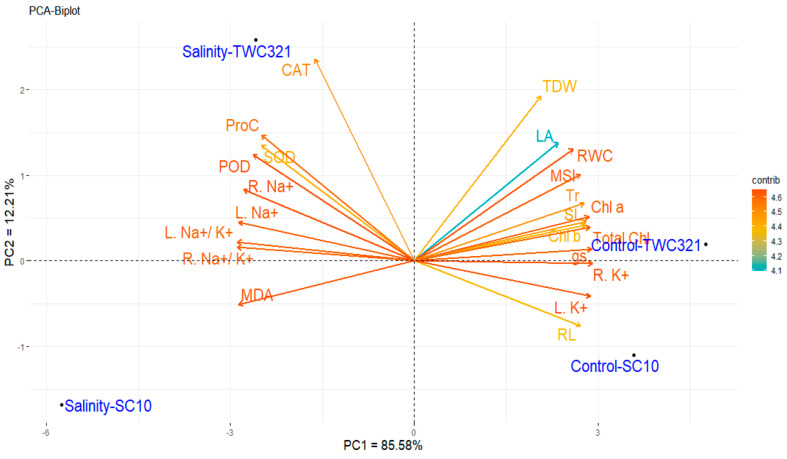
The biplot of principal component analysis shows the associations between studied characteristics and hybrids under salt-stressed and control conditions.

The association between assessed maize hybrids under salinity and non-stressed conditions and the evaluated parameters was explored utilizing principal component (PC) analysis. The first two PCs accounted for a considerable percentage of the total variance, with the first PC explaining 85.58% and the second PC capturing 12.21% (Figure 7). The first PC exhibited more significant variation, distinguishing the treatments from negative and positive values along the PC1 axis. The two hybrids under non-stress conditions (Control-SC10 and Control-TWC321) clustered on the right side of PC1, reflecting similar responses under non-stressed conditions, while the hybrids under salinity conditions (Salinity-SC10 and Salinity-TWC321) are separated on the left side, presenting the significant impacts in different responses of salinity stress. Under salinity stress, TWC321 is positioned closer to parameters associated with salinity tolerance, such as enzymatic antioxidants (SOD, POD, and CAT) and proline content (ProC). This proximity suggests that TWC-321 activates a stronger and more efficient antioxidant defense system against oxidative stress caused by salinity. Additionally, TWC321 is located farther from sodium-related parameters (root Na^+^, leaf Na^+^, and Na^+^ to K^+^ ratio), indicating better regulation of sodium uptake and improved ionic homeostasis under salinity conditions than SC10. Furthermore, SC10 is more associated with markers of stress damage (MDA), reflecting higher lipid peroxidation levels and cellular damage under salinity stress. Variables such as CAT, ProC, SOD, POD, leaf Na, root Na^+^, root Na^+^ to K^+^ ratio, and leaf Na^+^ to K^+^ ratio are associated with salinity stress conditions, indicating increased oxidative stress and improved activity of antioxidant defense mechanisms in both hybrids under salinity conditions. These stress-related variables demonstrated that the hybrids mitigate the damaging effects of salinity by elevating these parameters. In contrast, traits related to growth and photosynthesis, such as TDW, LA, RWC, Chl *b*, Chl *a*, and total Chl, were associated with non-stressed conditions, reflecting superior performance under non-stressed conditions. The analysis revealed that TWC-321, under salinity stress, was closely associated with key parameters linked to salt tolerance, including enzymatic antioxidants (SOD, POD, and CAT), proline content (ProC), and sodium regulation markers (Na^+^ to K^+^ ratio, root Na^+^, and leaf Na^+^). These parameters were positioned near TWC-321 on the PCA plot, highlighting its enhanced ability to mitigate oxidative stress and maintain ionic homeostasis under salt stress. In contrast, SC-10 was more closely associated with markers of stress damage, such as MDA, which indicates higher lipid peroxidation and cellular damage under salinity conditions. Additionally, traits related to growth and photosynthesis (e.g., TDW, LA, RWC, and chlorophyll content) were more closely associated with non-stress conditions.

**Figure 8:**
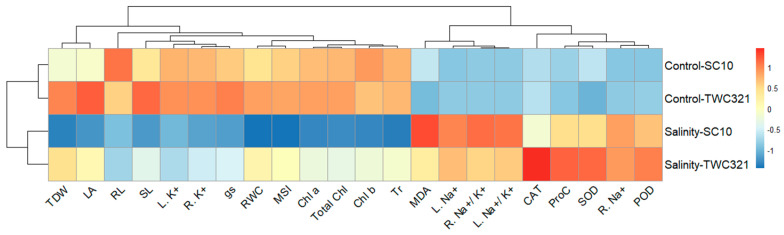
Heatmap showing the association between the studied characteristics and evaluated hybrids under salt-stressed and control conditions.

Furthermore, the heatmap cluster analysis (Figure 8) shows the correlation between SC-10 and TWC-321, and the analyzed traits, under salt-stressed and non-stressed conditions. Blue cells indicate low values and red cells present high values. The clustering of treatments revealed distinct patterns in two different clusters; the first group included the two hybrids under non-stressed conditions (Control-SC10 and Control-TWC321), while the other contained the two hybrids under salinity conditions (Salinity-SC10 and Salinity-TWC321). This separation displayed significant differences in hybrid responses under stress and non-stressed conditions. Additionally, the two hybrids showed comparable values for the main variables under non-stressed conditions. Nevertheless, TWC-321 showed higher values for all parameters except MDA, root Na^+^, leaf Na^+^, and Na^+^ to K^+^ ratio under salinity conditions, where lower scores indicate salinity tolerance.

### 3.8. Correlation Analysis

**Figure 9:**
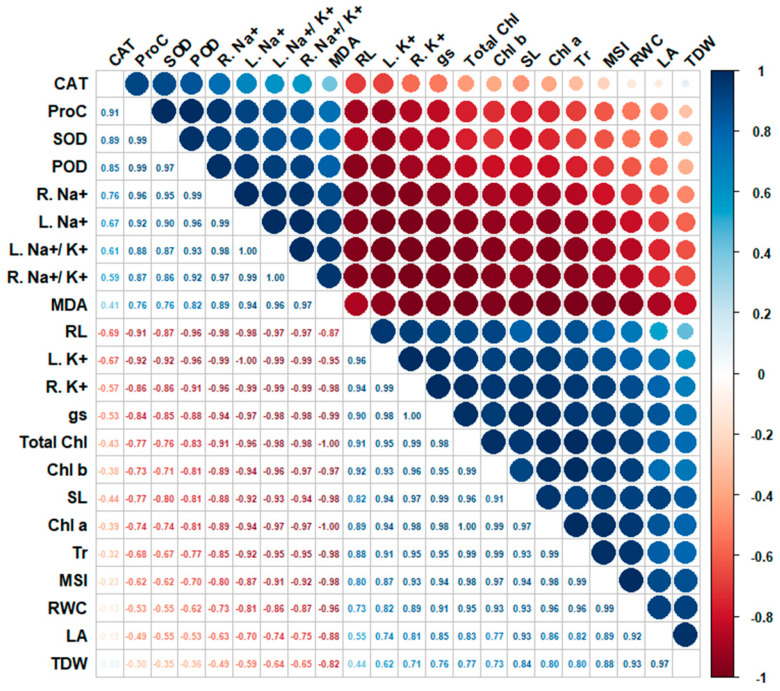
Spearman correlation matrix among the traits studied under salt stress conditions.

Figure 9 displays Spearman correlation coefficients between the characteristics under study under salinity stress. The correlation analysis was conducted using data from both maize hybrids under salinity stress conditions to explore the relationships between physiological and biochemical parameters. This approach provided a comprehensive understanding of how key traits are interrelated, despite the differing responses of the two hybrids to salt stress. Total dry weight (TDW) showed a high positive correlation with leaf area (LA) (r = 0.97), emphasizing the importance of leaf expansion in biomass accumulation. Similarly, total chlorophyll (Total Chl) strongly correlated with chlorophyll a and b (r = 0.99). Relative water content (RWC) also demonstrated a strong positive relationship with leaf area (r = 0.88), highlighting the role of water retention in supporting plant growth. Proline content also showed moderate positive correlations with biomass traits, including TDW (r = 0.57) and LA (r = 0.53). In contrast, malondialdehyde (MDA) negatively correlated with leaf area (r = −0.82), TDW (r = −0.82), and RWC (r = −0.85), indicating that oxidative damage hinders plant growth. Root sodium content (R. Na^+^) negatively correlated with root length (r = −0.87), and leaf sodium content (L. Na^+^) correlated negatively with RWC (r = −0.87). The Na^+^ to K^+^ ratio also negatively impacted photosynthesis. The total chlorophyll (r = −0.75) and chlorophyll b (r = −0.77) had a negative correlation with the root Na^+^ to K^+^ ratio (R. Na^+^/K^+^). Additionally, there was a negative correlation between MDA and potassium levels in roots (r = −0.93) and leaves (r = −0.92), indicating that potassium protects against oxidative damage.

## 4. Discussion

Understanding the mechanisms underlying salt tolerance in maize hybrids at the physiological, biochemical, and molecular levels is essential for improving crop resilience and advancing breeding strategies. This study compared the salt tolerance of two high-yielding Egyptian maize hybrids, SC-10 and TWC-321, under 200 mM NaCl stress using a comprehensive approach that included physiological, biochemical, transcriptional, and growth analyses. The results revealed distinct differences in the response of the hybrids to short-term salinity stress, with TWC-321 exhibiting superior tolerance compared to SC-10, as indicated by better growth and dry matter accumulation.

Salinity stress caused a significant increase in Na^+^ accumulation in both the roots and leaves of the hybrids. SC-10 displayed a 26.9% higher Na^+^ concentration in its roots compared to TWC-321, though no significant differences were observed in the leaves. The accumulation of Na^+^ is known to disrupt cellular homeostasis and impair plant growth, primarily through osmotic imbalance at the root–soil interface and subsequent depolarization of cell membranes [53]. Our findings suggest that TWC-321 has a more efficient Na^+^ exclusion or compartmentalization mechanism in its roots than SC-10, which could contribute to its better tolerance. This observation aligns with previous studies by Rizk et al. [15] and Huqe et al. [54], who reported significant variation in Na^+^ exclusion mechanisms among maize cultivars, supporting our observation that TWC-321 exhibits more efficient Na^+^ exclusion compared to SC-10. These studies highlight that efficient Na^+^ exclusion, particularly at the root level, is a key factor contributing to enhanced salt tolerance. In contrast, our results suggest that SC-10 has a less effective Na^+^ exclusion mechanism, which may explain its reduced tolerance to salinity. Furthermore, the higher Na^+^ levels in TWC-321 leaves point to vacuolar Na^+^ sequestration as a critical stress tolerance mechanism. This process is mediated by tonoplast Na^+^/H^+^ antiporters, which utilize the proton gradient to transport Na^+^ into vacuoles, reducing Na^+^ toxicity in the cytosol [55]. The ability of TWC-321 to exclude Na^+^ in the roots and sequester it in the leaves enables it to maintain ion balance, reduce toxicity, and sustain growth under saline conditions, distinguishing it from the more salt-sensitive SC-10.

Potassium (K^+^) is crucial for maintaining cell turgor, enzyme activity, and ion balance under stress. Salt stress often leads to an increase in Na^+^ and a decrease in K^+^, disrupting ion homeostasis [56,57]. In this study, K^+^ concentrations were significantly reduced in SC-10, with a 25.3% reduction in roots and an 18.74% reduction in leaves. In contrast, TWC-321 exhibited only an 18.27% reduction in roots, with no significant change in K^+^ levels in the leaves, suggesting that TWC-321 is more effective at maintaining K^+^ homeostasis under salt stress. Gene expression analysis supports this observation, with TWC-321 showing a greater upregulation of *zmHKT2* (1.96-fold) compared to SC-10 (1.42-fold). This upregulation indicates that *zmHKT2* plays a crucial role in Na^+^ exclusion in TWC-321, helping to reduce Na^+^ accumulation in the shoots and maintain ion balance. The differential expression of *zmNHX1*, with a 1.92-fold increase in TWC-321 and a slight decrease in SC-10 (0.8-fold), further emphasizes the superior salt tolerance of TWC-321. *zmNHX1* facilitates vacuolar Na^+^ sequestration, which helps maintain the optimal Na^+^/K^+^ ratio necessary for cellular function. These results align with Pitann et al. [37], who demonstrated that higher expression of NHX transporters, such as *zmNHX1*, is associated with better salt tolerance, which is consistent with our findings where TWC-321 showed a significant upregulation of *zmNHX1*. This indicates that higher NHX expression in tolerant maize varieties leads to improved Na^+^ exclusion and vacuolar Na^+^ sequestration.

It is important to acknowledge conflicting findings in the literature regarding salt tolerance mechanisms in maize. While our study emphasizes Na^+^ exclusion and vacuolar Na^+^ sequestration as key factors in the salt tolerance of TWC-321, some studies suggest that other mechanisms, such as enhanced antioxidant defense or specific ion transporters like Na^+^/H^+^ antiporters, may play a more prominent role in certain maize varieties. Kholova et al. [58] proposed that oxidative stress responses, including the activity of enzymes, are the primary determinants of salt tolerance rather than Na^+^ exclusion mechanisms. Similarly, studies by Shabala et al. [59] found that cultivars with high K^+^ retention under salt stress exhibited better growth, even in the presence of higher Na^+^ concentrations, suggesting that K^+^ homeostasis might be a more crucial factor than Na^+^ exclusion. These results highlight the complexity of salt tolerance mechanisms and suggest that tolerance may depend on a combination of factors, rather than a single mechanism.

Reactive oxygen species (ROS) are generated when Na^+^ toxicity disrupts cellular homeostasis, leading to membrane damage through lipid peroxidation. Malondialdehyde (MDA), a byproduct of lipid peroxidation, serves as an indicator of oxidative stress. In this study, MDA levels in SC-10 were twofold higher than in the control, while TWC-321 showed no significant change. This suggests that SC-10 experienced greater oxidative damage under salt stress, as indicated by the elevated MDA concentrations. These results align with similar studies in rice, where salt-tolerant genotypes exhibited lower MDA levels and reduced oxidative damage [60,61]. Therefore, MDA content could serve as a key marker for identifying salt-tolerant genotypes.

Antioxidant enzymes such as SOD, POD, and CAT are critical for mitigating ROS-induced damage and enhancing stress tolerance. In this study, the activities of these enzymes increased significantly in TWC-321, suggesting a strong antioxidant defense system that helps protect against oxidative damage. In contrast, SC-10 showed low SOD and POD activity, with no significant change in CAT activity, indicating a weakened antioxidant response under salt stress. This finding supports the conclusion that the antioxidant defense system in TWC-321 is more effective in scavenging ROS and minimizing oxidative damage, contributing to its higher salinity tolerance. Similar observations have been reported in other salt-tolerant maize, barley, and rice cultivars [15,61,62].

Chlorophyll content, which is vital for photosynthesis, was significantly reduced in both hybrids under salt stress. However, TWC-321 exhibited a less pronounced decline in chlorophyll levels compared to SC-10, which could be attributed to its ability to exclude Na^+^ from the leaves. Excess Na^+^ accumulation can disrupt chlorophyll biosynthesis and accelerate its degradation [63], thereby impairing photosynthesis. These results are consistent with studies showing that salt-tolerant maize genotypes maintain higher chlorophyll content under salt stress [64]. The reduction in stomatal conductance and the transpiration rate observed in both hybrids under salt stress indicates a strategy of water conservation. However, TWC-321 maintained a better balance between water conservation and gas exchange, allowing for continued photosynthesis despite the stress.

Proline, an important osmoprotectant and antioxidant, accumulated to higher levels in TWC-321 than in SC-10 under salt stress. Proline helps to protect cellular membranes from oxidative damage and stabilizes proteins [65]. The increased proline levels in TWC-321 likely contributed to its lower MDA levels and enhanced salinity tolerance. The observation that proline accumulation is more pronounced in TWC-321 is similar to the findings by Rizk et al. [17], further emphasizing the role of osmoprotectants in enhancing stress resilience.

Multivariate techniques like principal component analysis (PCA) are particularly effective in identifying patterns within datasets involving complex variables [66,67,68]. Graphical representation of PCA loadings is an easy way to explore similar and different physiological reactions to environmental stresses [69,70,71]. PCA further confirmed the superior salt tolerance of TWC-321. The PCA loadings revealed that TWC-321 exhibited higher levels of proline, antioxidant defense (SOD, POD, and CAT), and better ion regulation, aligning with findings from Omar et al. [70]. These traits are critical for salinity tolerance and could be used for screening maize hybrids for salt stress tolerance at the seedling stage. These findings align with previous findings of Patwa et al. [72], highlighting the critical role of these traits in conferring salinity tolerance. Therefore, these characteristics could be helpful for fast-screening maize hybrids for salinity stress at the seedling stage.

## 5. Conclusions

The two evaluated maize hybrids were affected by salt stress, but TWC-321 demonstrated greater tolerance to salinity stress than SC-10. The mechanisms underlying TWC-321’s salinity tolerance likely include (i) the ability to maintain efficient Na^+^ exclusion in roots and vacuolar Na^+^ sequestration in the leaves, (ii) sustaining higher photosynthetic activity, and (iii) enhanced activity of antioxidant enzymes along with increased proline accumulation. These traits suggest that TWC-321 is a strong candidate for inclusion in maize breeding programs to improve sustainability in maize production in salt-affected soils. Further research is necessary to pinpoint the specific genes and molecular pathways that confer the enhanced salinity tolerance observed in TWC-321.

## Figures and Tables

**Table 1:** Sequence of the primers used for quantitative real-time RT-PCR.

Genes	Gene ID	Forward	Reverse
*zmHKT2*	XM_008646809	5′-GCTCAACTTCTCCACGTTCA-3′	5′-CACCACCCAGAGAAGCTGTA-3′
*zmHKT1;5*	NM_001175105	5′-TCAACTTCAGCGTCCTCAACA-3′	5′-GAATCCCACGTTGCCATACG-3′
*zmNHX1*	AY270036	5′-ATGCAGGGTTCCAAGTGAAG-3′	5′-AATATTGCCCCAAGTGCAAG-3′
*zmActin*	NM_001155179	5′-GACCTCACCGACCACCTAATG -3′	5′-CTGAACCTTTCTGACCCAATG-3′

## Data Availability

Data are contained within the article.
